# Comparison of Genetic Features and Evolution of Global and Chinese Strains of Community-Associated Methicillin-Resistant Staphylococcus aureus ST22

**DOI:** 10.1128/spectrum.02037-21

**Published:** 2022-02-09

**Authors:** Wangxiao Zhou, Ye Jin, Xiang Liu, Yunbo Chen, Ping Shen, Yonghong Xiao

**Affiliations:** a State Key Laboratory for Diagnosis and Treatment of Infectious Diseases, National Clinical Research Center for Infectious Diseases, Collaborative Innovation Center for Diagnosis and Treatment of Infectious Diseases, The First Affiliated Hospital, Zhejiang University School of Medicine, Hangzhou, China; University of Calgary

**Keywords:** EMRSA-15, ST22-MRSA, genomic evolution, virulence, *agr*

## Abstract

Methicillin-resistant Staphylococcus aureus (MRSA) sequence type (ST) 22, especially the epidemic MRSA-15 (EMRSA-15), has been one of the most important disease-causing clones transmitting rapidly within and between hospitals globally. However, the genetic features and evolution of Chinese MRSA ST22 remain to be determined. Herein, we performed comparative genomics analysis of 12 ST22 community-associated (CA) MRSA isolates from China with 9 Chinese ST22 CA-MSSA isolates and 284 ST22 genomes from global sources, to clarify the genotypic features and potential transmission of MRSA ST22 strains isolated in China. Phylogenetic reconstruction and time estimation suggested that the Chinese subclade emerged around 2006, and the ST22-SCC*mec* V clone may have evolved from the native ST22-MSSA clone rather than spread from other regions, indicating that the Chinese ST22-MRSA-V clone is independent of the EMRSA-15 and Gaza clone, with differences in *lukSF*-*PV* and *tsst-1* carriage. Virulence assays suggested that the ST22-MRSA clone was highly virulent, displaying higher or similar virulence potential as MSSA ST22 predecessors and the epidemic USA300 and ST22-MSSA. However, two nonsense mutations caused by a frameshift in *agrC* were identified in two ST22-MSSA isolates, resulting in a significant attenuation of virulence. RT-qPCR also demonstrated that the high virulence potential of these ST22 strains may be attributed to elevated expression of *agr*. This study provides insight into the epidemiology of the novel and highly virulent CA-MRSA ST22 clones.

**IMPORTANCE** Staphylococcus aureus sequence type 22 (ST22) is the main HA-MRSA clone spreading in Europe. It has strong capacity to supplant and replace other formerly epidemic MRSA clones. Previous work has described genotypic characteristics of ST22 belonging to EMRSA-15 and Gaza clone; however, the genetic feature and virulence potential of Chinese spread of ST22 strains are still limited. We conducted a detailed analysis of genomic evolution of global ST22 strains, to clarify the genotypic features and potential transmission of MRSA ST22 strains isolated from China. Our results suggested that the Chinese subclade is highly virulent, and emerged around 2006. We also demonstrated that the ST22-SCC*mec* V may have evolved from the native ST22-MSSA clone rather than spread from other regions, and the high virulence potential of these ST22 strains may be attributed to the high expression of *agr* based on the results of virulence assays of Chinese ST22 clones. Our findings are of great importance for providing insights into the epidemiology and pathogenicity of global and Chinese ST22 clones.

## INTRODUCTION

Since first reported in 1961, methicillin-resistant S. aureus (MRSA) has become a public health priority and major cause of infections in most health care settings and community settings worldwide. The prevalence and the epidemiology of MRSA are in a constant state of change, with newly adapted emerging MRSA clones predominating in different geographical regions. Furthermore, molecular epidemiology has demonstrated that some MRSA clones are prevalent in geographically limited regions, while others can spread worldwide (1). However, the changes in the prevalence and the epidemiology of different MRSA clones among different geographical regions are still unknown.

ST22 is a successful methicillin-resistant Staphylococcus aureus (MRSA) lineage identified in hospitals. The epidemic MRSA (EMRSA)-15 (ST22-IVh) strain (2) is the most rapidly transmitted hospital-acquired MRSA clone across Europe and in other continents (3, 4), including Asian countries such as Singapore and India (5, 6). ST22-MRSA has strong capacity to supplant and replace other formerly epidemic MRSA clones (7–9). Of concern is the increasing emergence of severe community-associated (CA) infections caused by ST22-MSRA containing the *lukS*/*F* genes (encoding the Panton-Valentine leucocidin) (10, 11).

Although frequently isolated in many countries, in China, ST22-MRSA is still a sporadic MRSA clone. Our group has isolated a total of 749 MRSA strains from various provinces and cities in China, and detected 12 ST22 CA-MRSA isolates, which caused severe bloodstream infections (12). Recent studies have reported the emergence of the CA-methicillin-susceptible S. aureus (MSSA) ST22-PVL+ clone in China (13–15); however, detailed information is still limited. Therefore, to describe the genotypic characteristics and possible transmission of CA-MRSA ST22 strains isolated from China, we performed detailed phenotypic and comparative genomic analyses between 12 MRSA ST22 isolates and 9 MSSA ST22 isolates from our database and 284 ST22 isolates from the NCBI.

## RESULTS

### General characteristics of the global ST22 clones.

**FIG 1:**
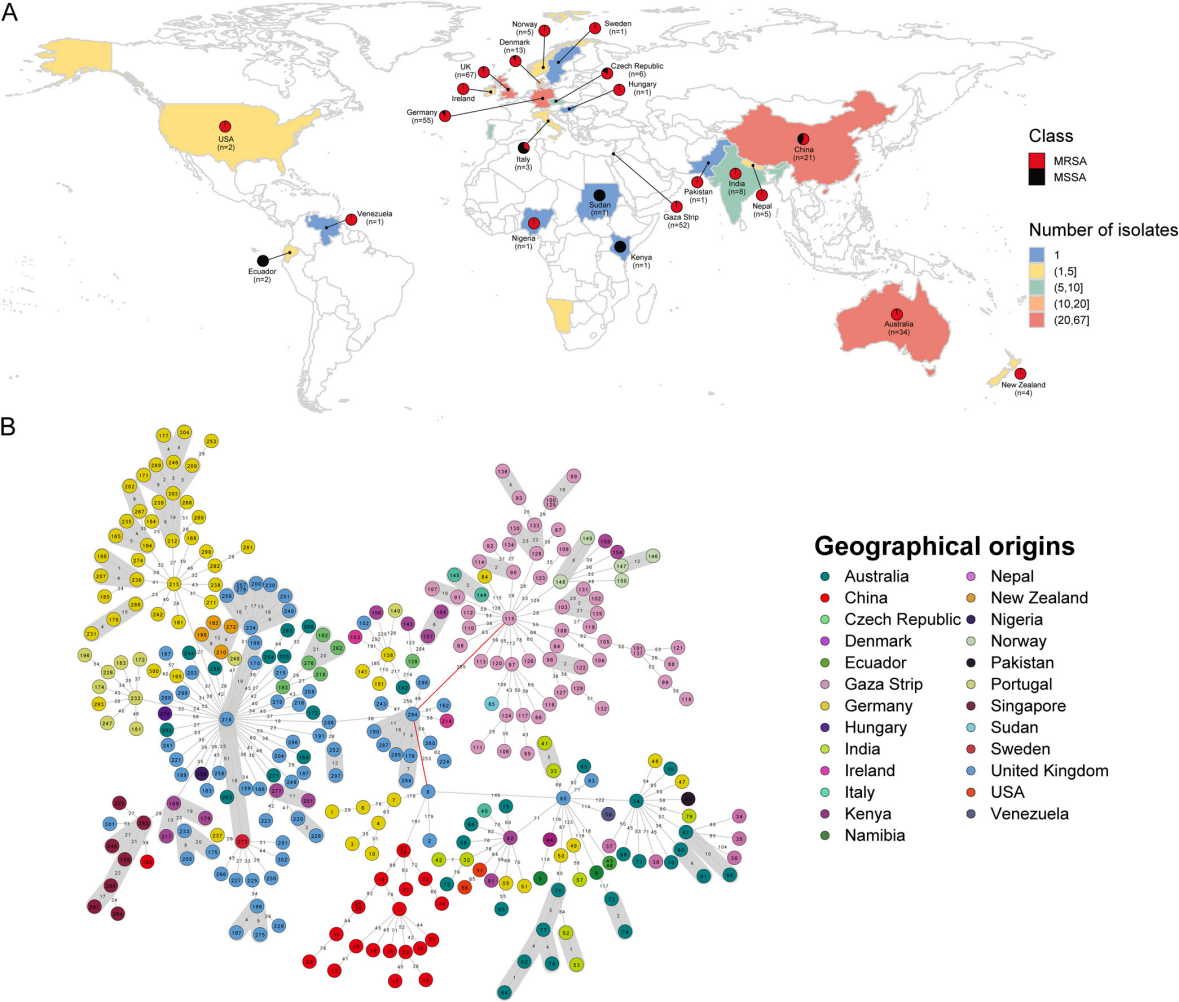
Relationships among 305 ST22 isolates in this study. (A) Geographical distribution of 305 ST22 S. aureus isolates. Red and black dots represent MRSA and MSSA isolates, respectively. The countries with ST22 isolates are marked by background color fill. (B) MST of 305 ST22 isolates. Each circle represents a ST22 isolate, with filled colors based on geographic origin. The connecting lines indicate the number of SNPs in a pairwise comparison. The transmission cluster is marked in gray based on the threshold of 23 SNPs.

To characterize the genetic features of ST22 MRSA strains isolated from China, we analyzed publicly available ST22 MRSA genomes (*n* = 264) from 24 regions and 12 additional ST22 MRSA genomes from our collection, with ST22 MSSA strains used for comparison (20 from the NCBI database and 9 from our collection). The 12 Chinese ST22 MRSA isolates (11 of ST22-SCCmec V and 1 of SCCmec IVh) were isolated from severe CA-MRSA infection cases at different Chinese hospitals, and other global ST22 clones were obtained from Australia, Italy, the United Kingdom, Gaza Strip, and other countries, with widespread geographic distributions (Fig. 1A). Among these ST22 clones, we defined the number of single nucleotide polymorphisms (SNPs) separating isolates to identify possible transmission pairs, together with epidemiological investigation. As shown in Fig. 1B, SNPs of isolates in ST22 clones were from 0 to 452, and the SNPs of gray area in ST22 clones were <24 SNPs, which indicated that these strains were closely related.

**FIG 2:**
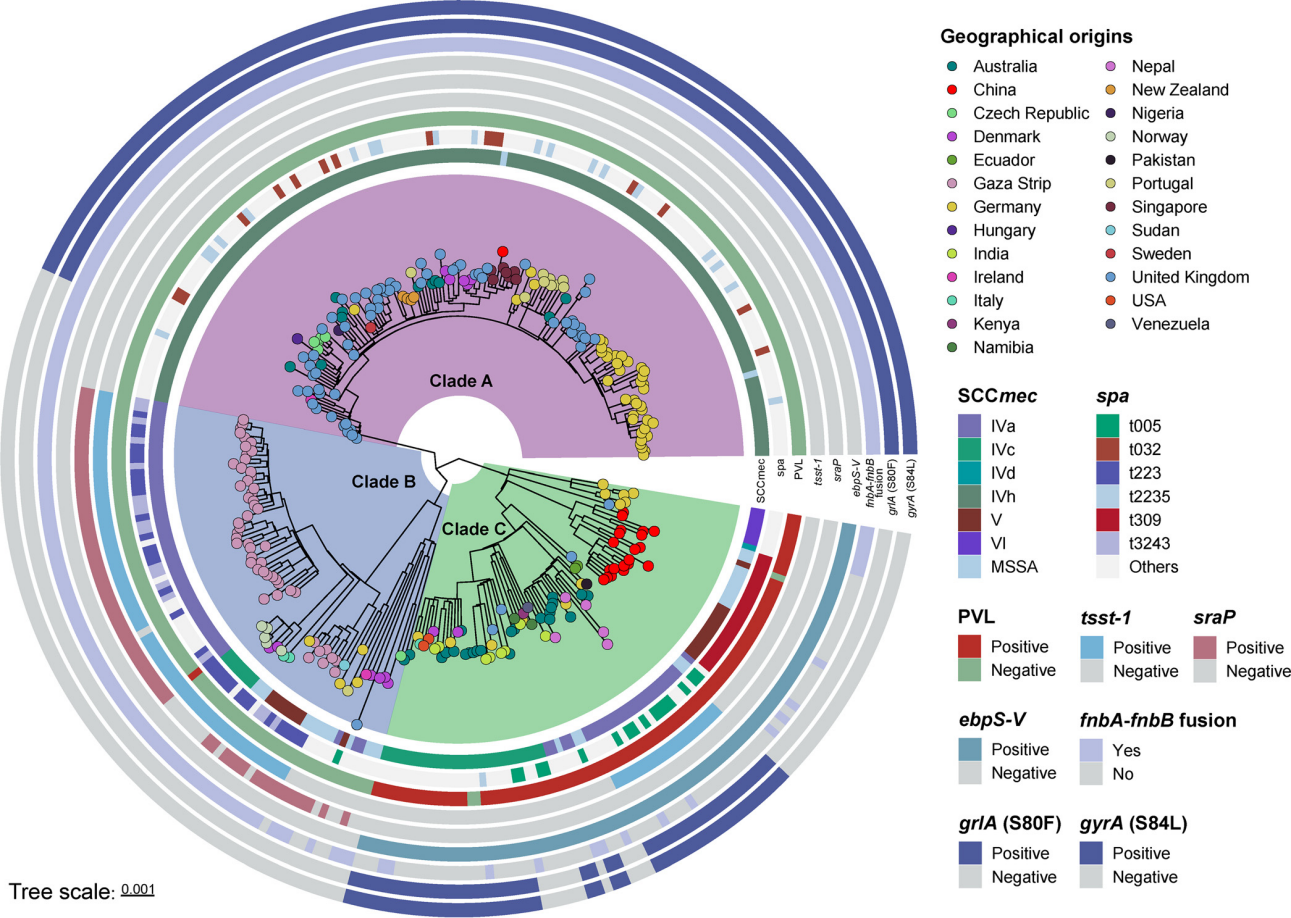
Phylogenetic structure of 305 ST22 strains. The SCC*mec* type, *spa* type, presence of PVL, *tsst-1*, *sraP*, *ebps-V*, *fnbA*-*fnbB* fusion, and quinolone resistance-determining region (QRDR) mutations (*grlA* and *gyrA*) are mapped on the tree (from inner to outer circle).

The 305 ST22 genomes were divided into three main clades (Fig. 2). The largest clade, clade A, contained 147 genomes, mostly originating from the United Kingdom, and other regions such as Germany and Singapore. In this clade, all ST22 MRSA isolates harbored SCC*mec* IVh, belonging to EMRSA-15. Notably, one ST22 MRSA strain (ST22-IVh) from China was found in clade A and gathered with strains from Singapore. Clade B was represented by the Gaza clone, which was mainly isolated from the Gaza Strip. In this clade, almost all ST22 MRSA isolates carried SCC*mec* IVa. Clade C can be divided into three subclades (CI, CII, and CIII) and two singletons, with clade CII harboring the largest number of genomes (*n* = 20) from China. Almost all the ST22 MRSA isolates from China (11/12) belonged to SCC*mec* V, and in the whole phylogenetic tree, the *spa* type t309 was found only in ST22 clones in China, whereas the ST22-SCC*mec* IVh clone from China belonged to t032. These results suggested that the “China clone” may be divided into two distinct lineages resulting from two separate acquisitions of the SCC*mec* elements.

**FIG 3:**
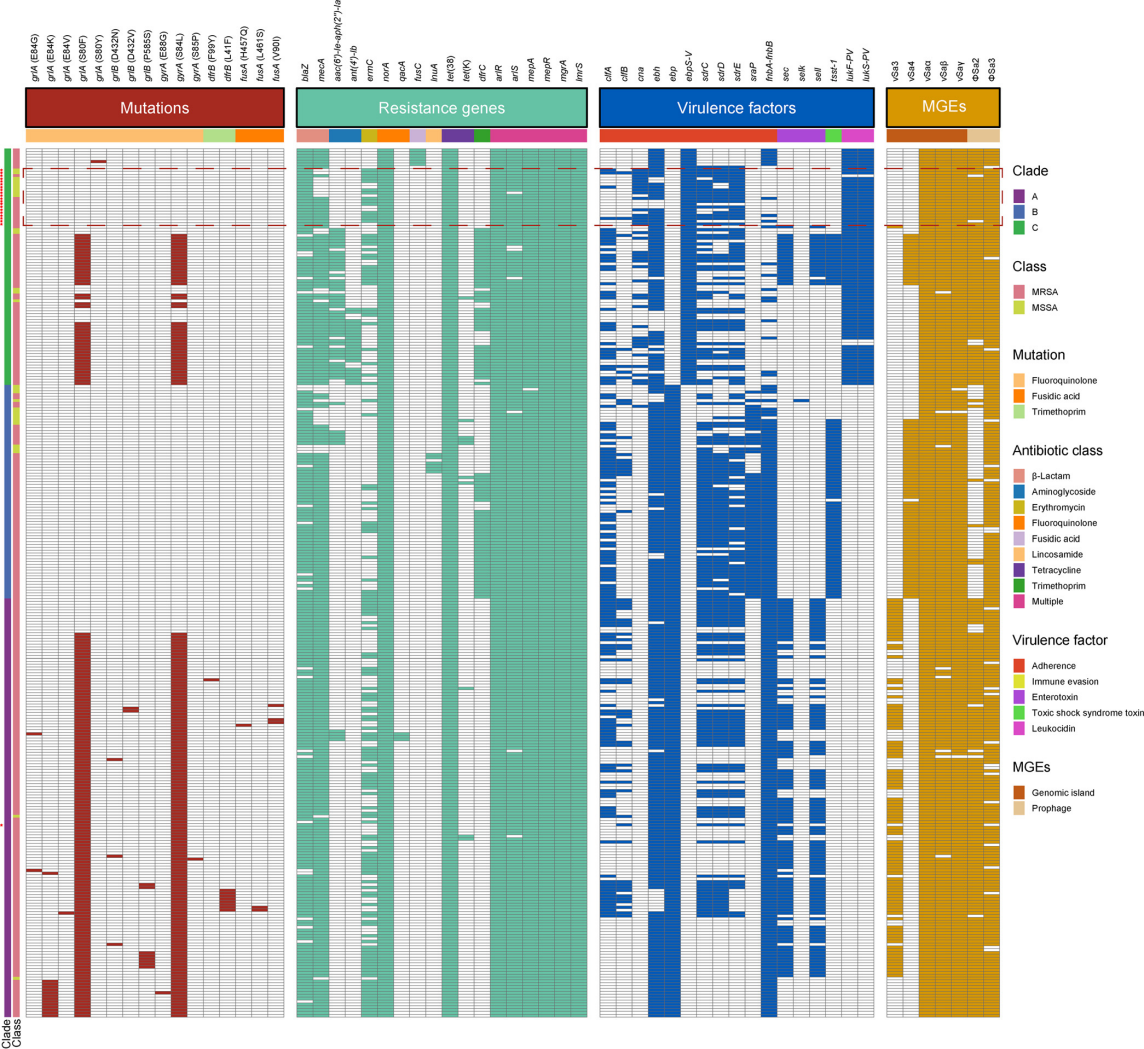
Distributions of mutations, antimicrobial resistance genes, virulence factors, and MGEs in the ST22 isolates. The isolates from China are indicated by red asterisks and boxes.

To compare ST22-MRSA isolates in China with those from other regions, we investigated the presence of virulence genes. As shown in Fig. 3, none of the ST22-MRSA strains from clade A contained *tsst-1*, while almost all clones of clade B (53/54) harbored *tsst-1*. However, all strains of clade C isolated from China contained PVL (except SKLX100539), while clades A and B did not. In addition to the mobile genetic element (MGE)-induced difference of virulence genes in the genomic architecture of ST22 isolates, most Chinese ST22 isolates (17/21) carried the integrated fibronectin-binding protein (FnBP) locus, while all genomes of clade A and most of B (93.33%, 70/75) had a 2268-bp deletion in the FnBP locus.

To understand the different resistance patterns among the global ST22 strains, we evaluated the presence of resistance genes. There were no significant differences among clades A to C; however, mutations in the *grlA* and *gyrA* genes were present in most strains of clade A and clade C excluding Chinese ST22 clones (Fig. 3). It is important to emphasize that the predicted genotypes (resistance to fluoroquinolones) of the ST22-SCC*mec* IVh clone from China (SKLX111090) were consistent with the result of antimicrobial susceptibility testing (Table 1).

**TABLE 1:** Antimicrobial susceptibility of 21 ST22 isolates from China^*a*^

Strains	ERY	CLI	OXA	Pen	SXT	TCY	VAN	RIF	CIP	LVX	MFX	GEN	AMK	TGC	LNZ	DAP
SKLX100316	R	R	R	R	S	S	S	S	S	S	S	S	S	S	S	S
SKLX100539	R	R	R	R	S	S	S	S	S	S	S	S	S	S	S	S
SKLX101489	S	S	R	R	S	S	S	S	S	S	S	S	S	S	S	S
SKLX102523	R	R	R	R	S	S	S	S	S	S	S	S	S	S	S	S
SKLX108615	R	R	R	R	S	S	S	S	S	S	S	S	S	S	S	S
SKLX111090	S	S	R	R	S	S	S	S	R	R	R	S	S	S	S	S
SKLX116135	R	R	R	R	S	S	S	S	S	S	S	S	S	S	S	S
SKLX119044	R	R	R	R	S	S	S	S	S	S	S	S	S	S	S	S
SKLX119055	R	R	R	R	S	S	S	S	S	S	S	S	S	S	S	S
SKLX119058	R	R	S	R	S	S	S	S	S	S	S	S	S	S	S	S
SKLX41347	R	R	S	R	S	S	S	S	S	S	S	S	S	S	S	S
SKLX55947	S	S	S	R	S	S	S	S	S	S	R	S	S	S	S	S
SKLX55948	S	S	S	R	S	S	S	S	S	S	S	S	S	S	S	S
SKLX56567	R	R	S	R	S	S	S	S	S	S	S	S	S	S	S	S
SKLX56568	R	R	S	R	S	S	S	S	S	S	S	S	S	S	S	S
SKLX57091	S	S	S	R	S	S	S	S	S	S	S	S	S	S	S	S
SKLX59047	R	R	S	R	S	S	S	S	S	S	S	S	S	S	S	S
SKLX60562	R	R	R	R	S	S	S	S	S	S	S	S	S	S	S	S
SKLX61193	R	R	S	R	S	S	S	S	S	S	S	S	S	S	S	S
SKLX80731	S	S	R	R	S	S	S	S	S	S	S	S	S	S	S	S
SKLX83059	S	S	R	R	S	S	S	S	S	S	S	S	S	S	S	S

a R, resistant; S, susceptible; ERY, erythromycin; CLI, clindamycin; OXA, oxacillin; PEN, penicillin G; SXT, trimethoprim-sulfamethoxazole; TCY, tetracycline; VAN, vancomycin; RIF, rifampin; CIP, ciprofloxacin; LVX, levofloxacin; MFX, moxifloxacin; GEN, gentamicin; AMK, amikacin; TGC, tigecycline; LNZ, linezolid; DAP, daptomycin.

### Phylogeographical context and comparative genomics of ST22 strains.

**FIG 4:**
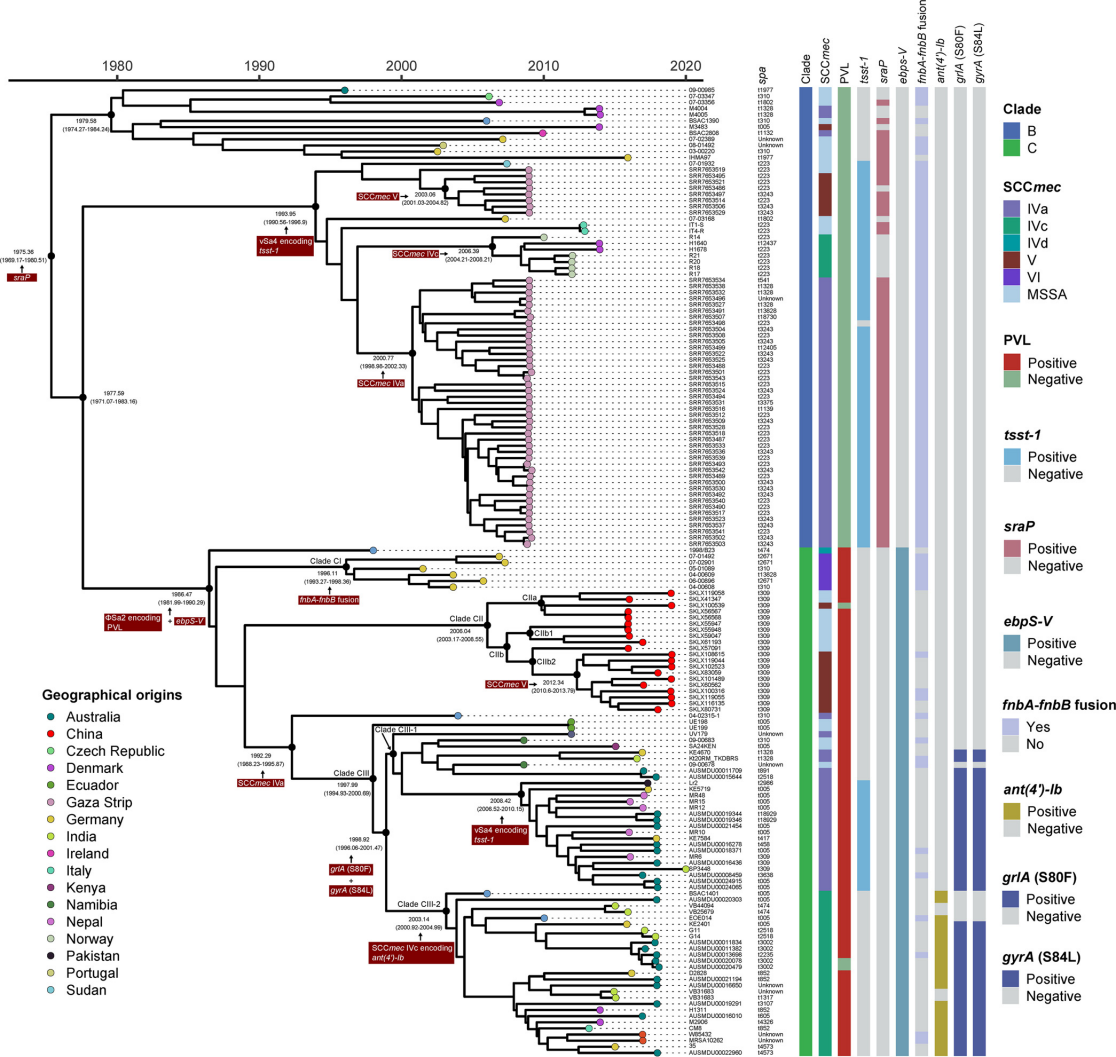
Time-based tree of the 158 non-ST22-A strains. The characteristics of each strain are shown on the right, including clades, type of SCC*mec*, presence of PVL, *tsst-1*, *sraP*, *ebps-V*, fusion of *fnbA*-*fnbB*, *ant(4′)-Ib*, and (QRDR) mutations (*grlA* and *gyrA*). The geographical origin of isolates is mapped on the tips. Main evolutionary events are exhibited in red boxes, with relevant divergence time and 95% HPD intervals shown at the nodes.

Next, we carried out a Bayesian phylogenetic estimation to investigate the global evolutionary history of Chinese ST22-MRSA clone and Gaza clone belonging to non-ST22-A. The Gaza clone (clade B) possibly emerged in ∼1975, while the clade C emerged in ∼1986 (Fig. 4). BactDating estimated that *sraP* integrated in clade B ST22 clones was in ∼1975. Then in 1994, the vSa4 (containing *tsst-1*) was integrated in clade BII.

None of the ST22 isolates in clade B contained PVL, while the ΦSa2 (containing PVL) integrated in ST22 clones in clade C emerged in ∼1986. Furthermore, our analysis divided clade C into three main clades (I, II, and III) (Fig. 4), and our ST22 isolates were gathered in clade CII that emerged in ∼2006. The mapping of ST22 strains showed the independent evolution of ST22 MRSA in clade CII, indicating that the Chinese ST22 lineage (clade CII) was a new subtype that differed from other ST22-SCC*mec* V clones in different regions. Notably, clade CII clones could be further divided into two subclades (CIIa and CIIb), with clade CIIb2 containing almost all ST22 MRSA strains (10/12) from China. Separation of clade CIIb1 from CIIb2 correlated with the acquisition of SCC*mec* V. An ST22 MSSA isolate (SKLX57091) was found in CIIb and was more closely related to all MRSA ST22 strains than other MSSA strains in CIIb. Similarly, a MRSA isolate (SKLX100539) found in clade CIIa was surrounded by all MSSA isolates of clade CIIa, indicating that the Chinese MRSA ST22 clones probably arose from ST22-MSSA isolates, independent of the EMRSA-15 and Gaza clone.

### Comparison of Staphylococcal genomic islands in ST22 genomes.

**FIG 5:**
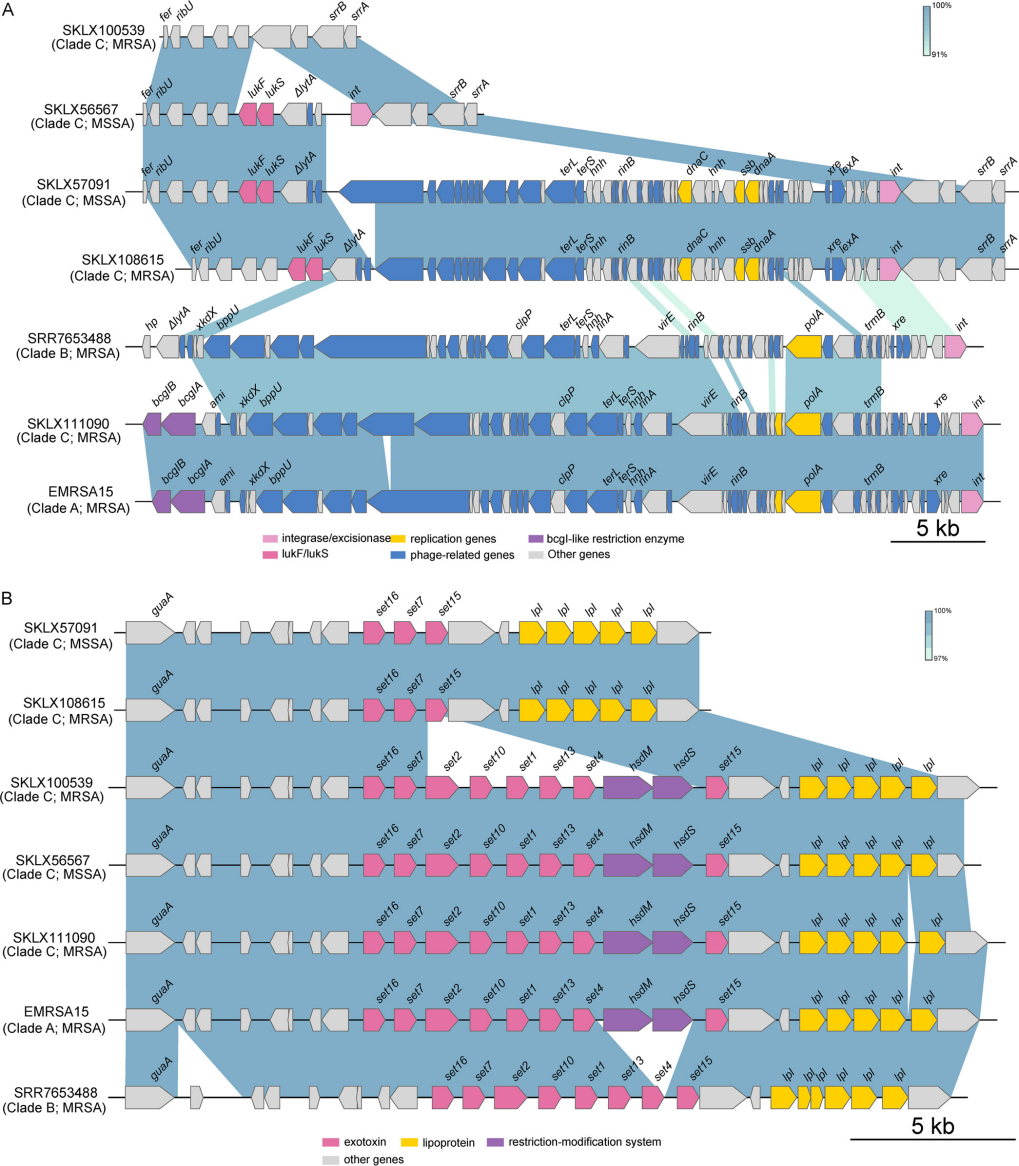
Comparison of ΦSa2 (A) and vSaα (B) between ST22 strains in each clade. Genes are indicated by arrowed boxes and colored based on gene function classification.

To understand the differences in the Staphylococcal genomic island and prophages among three clades in ST22 isolates, we analyzed the presence and sequence variations in all ST22 genomes. All ST22 isolates carried a ΦSa2 structure, but only the clade C isolates contained ΦSa2 encoding PVL (Fig. 5A). The sequence alignment showed that, compared with a PVL negative ST22 MRSA strain (SKLX100539) in clade C, an ΦSa2 containing PVL with variable size (from ∼6.7–37 kb) was inserted upstream of the riboflavin transporter (RibU) and downstream of the staphylococcal respiratory response AB regulator (SrrAB) in other PVL-positive clade C isolates (Fig. 5A). However, compared with clade A, ΦSa2 in clade B lacked the *bcgIAB*, which encodes a BcgI-like restriction enzyme.

All clades harbored vSaα, which consisted of a cluster of tandem staphylococcal exotoxin (*set*) genes, the glutamine synthase gene (*guaA*), lipoprotein genes (*lpl*), and a restriction/modification system (*hsdM*/*hsdS*). The structure of vSaα in Chinese ST22 MRSA isolates of clade CIIb2 (e.g., SKLX108615) was consistent with that of ST22-MSSA (SKLX57091) (Fig. 5B). The vSaα in SKLX108615 and SKLX57091 only harbored *set7*, *set1*5, and *set16*. However, the structure of vSaα in another Chinese ST22 MRSA isolate (SKLX100539) harbored an intact set gene cluster and the restriction/modification system (*hsdM*/*hsdS*), which was identical with that of the ST22-MSSA strain of CIIa (e.g., SKLX56567). However, the structure of vSaα in the ST22-MRSA-IVh isolate (SKLX111090) d EMRSA-15 showed the same pattern. Therefore, combined with the results of the phylogenetic analysis, we speculated that the Chinese ST22 MRSA clones that harbored unique SCC*mec* V may have evolved from methicillin-susceptible ST22 predecessors that acquired resistance to methicillin.

### Virulence of Chinese ST22-MRSA isolates in this study.

**FIG 6:**
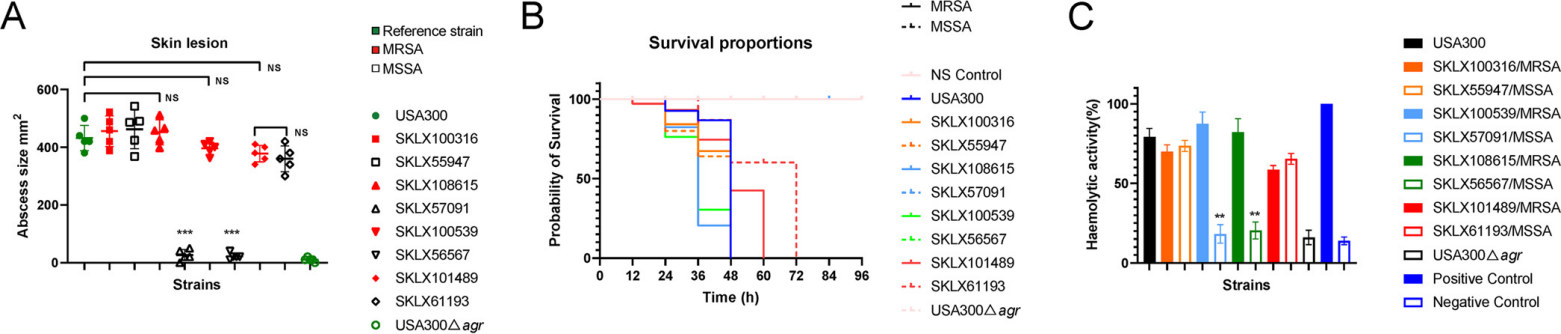
Virulence phenotype of selected ST22 isolates from China, the CA-MRSA clone USA300 and its Δ*agr* mutant strain USA300Δ*agr*. (A) Skin lesion in the mouse skin infection model. Five mice were infected per isolate. (B) Kaplan–Meier survival estimates for mice (*n* = 10 per isolate) injected with 2 × 10^8^ CFU of isolates or NaCl solution. (C) Hemolytic capacity estimates of selected isolates. Purified water was used as positive control, and rabbit RBCs resuspended in NaCl solution were used as negative control. **, *P < *0.01; ***, *P < *0.001; NS, not significant (*P ≥ *0.05).

To determine the virulence potential of ST22-MRSA *in vivo*, mouse models of skin infections and bloodstream infections were constructed. The closely related MSSA strains and the CA-MRSA clone USA300 famous for its hypervirulence, and its Δ*agr* mutant strain (USA300Δ*agr*), were used for comparison. Most ST22-MRSA and ST22-MSSA isolates produced significantly larger lesions than USA300Δ*agr* (*P < *0.001), but at levels comparable to USA300 (*P > *0.05) (Fig. 6A and Table S2 in the supplemental material), indicating that both ST22-MRSA and ST22-MSSA were hypervirulent. However, the skin lesions of ST22-MRSA isolates SKLX108615 and SKLX100539 were much larger than that of the closely related ST22-MSSA clones SKLX57091 and SKLX56567 (*P < *0.001), respectively. In bloodstream infection models, as shown in Fig. 6B and Table S3, mice infected with ST22-MRSA strains and ST22-MSSA strains (except SKLX57091 and SKLX56567) all died within 72 h (*n* = 10), while those infected with the closely related ST22-MSSA strains SKLX57091 and SKLX56567 were still alive until euthanasia. To further investigate the virulence of ST22-MSSA strains SKLX57091 and SKLX56567, we then compared the alpha-toxin activity by analyzing lysis of rabbit RBCs. Alpha-toxin activity often represents *agr* expression in strains. As shown in Fig. 6C and Fig. S1, we found that all ST22 strains displayed much higher hemolytic capacity than ST22-MSSA clones SKLX57091 and SKLX56567 (*P < *0.01).

### *agr* dysfunction accounts for the low virulence of ST22-MSSA.

We next compared the sequence of *agr* in ST22-MRSA and ST22-MSSA. In ST22-MSSA SKLX57091 and SKLX56567, two nonsense mutations caused by frameshift in *agrC* (c.349delT and c.1140insT, respectively) were identified (Fig. S2A). These mutations accounted for the defect in *agr* expression and the avirulence of the strains. We next performed RT-qPCR to evaluate the expression of *hla* and *agrC*, which regulate toxins such as alpha-toxin and PSM*α* that are most frequently associated with CA-MRSA virulence among ST22-MRSA and ST22-MSSA isolates. All *hla* and *agrC* genes were highly expressed in ST22 isolates except SKLX57091 and SKLX56567 (*P < *0.01, Fig. S2B). Thus, the *agr* dysfunction may be responsible for the low virulence of the SKLX57091 and SKLX56567.

## DISCUSSION

MRSA ST22, especially the EMRSA-15, has spread across most countries, especially in Australia and the United Kingdom (4, 16–18). However, as a sporadically occurring MRSA clone in China, the genomic features and clonal origin of ST22-MRSA have not been fully identified. Therefore, herein, we characterized 12 ST22-MRSA isolates, which cause life-threatening infections.

We separated the ST22-MRSA isolates from China as two clones: t309-carrying SCC*mec* V clone and t032-carrying SCC*mec* IVh clone. The ST22-SCC*mec* V clone was resistant to only β-lactams (penicillin and oxacillin) and contained only *blaZ* and *mecA* genes. However, the ST22-SCC*mec* IVh clone was also resistant to fluoroquinolones. Phylogenetic reconstruction and time estimation suggested that the Chinese subclade emerged around 2006, and ST22-SCC*mec* V isolates may have evolved from the native ST22-MSSA rather than spreading from other regions to China. This indicated that the Chinese ST22-MRSA-V clones are independent of the EMRSA-15 and Gaza clone, with differences in PVL and *tsst-1* expression.

Our results suggested that the “Chinese clone” consists of two distinct lineages resulting from two separate acquisitions of the SCC*mec* cassettes. The acquisition of SCC*mec* V cassettes was a critical genetic event in the evolution of the Chinese ST22-MRSA clone resistant to β-lactam antibiotics. Notably, compared with MSSA strains, the larger SCC*mec* cassettes in MRSA strains have been reported to reduce the bacterial toxicity due to the expression of *mecA*, which can repress the expression of cytolytic toxins (19). Collins et al. (20) reported that this significant toxin repression could compensate for the energy-consuming maintenance of a large SCC*mec* element and its associated resistance to methicillin, but probably leads to reduced fitness outside of hospital settings. However, the virulence assays suggested that ST22-MRSA shows a level of virulence comparable to that of ST22-MSSA. *mecA* can inhibit *agr* expression; however, He et al. (21) have reported that the evolution of CA-MRSA clones is always accompanied by a very low methicillin resistance level. This may be associated with the reservation of the high virulence level of the MSSA predecessors of CA-MRSA. In this study, the MIC of oxacillin in ST22 is 4 mg/L, which is a breakpoint that served to classify MRSA. Therefore, our results highlight that the evolution of ST22 MSSA into ST22 MRSA was accompanied by low methicillin resistance level.

S. aureus produces many secreted virulence molecules, including the most prominent pore-forming toxins Hla, PSM*α*, and PVL, known to contribute to skin and soft infections, sepsis, and pneumonia (22). The production of these toxins is strictly controlled by global virulence regulatory system *agr*, and the occurrence of mutations in *agr* affect the ability of S. aureus to cause human infections (23, 24). In the present study, we identified the ST22-MRSA clone and its closely related ST22-MSSA from China, which have the same genetic features (ST22, t309, and identical virulence and resistance profile), displaying completely different virulence phenotypic response. The ST22-MRSA isolates were hypervirulent while the ST22-MSSA showed a very low level of virulence. Of note, two nonsense mutations caused by frameshift in *agrC* (c.349delT and c.1140insT, respectively) were observed in two ST22-MSSA isolates, which resulted in a significant attenuation of virulence. Thus, our study emphasizes the great importance of *agr* expression in maintaining the high toxicity of MSSA predecessors during CA-MRSA evolution.

In conclusion, this study provided the first detailed characterization of ST22-CA-MRSA strains. We determined that the ST22-MRSA-V clones from China are independent of the EMRSA-15 and Gaza clones. We also investigated the evolutionary history of a new and highly virulent ST22-MRSA clone, and revealed it belonged to sporadic MRSA clones that are rare and not epidemic in China. Nonetheless, sporadic MRSA isolates may constitute a significant extensive reservoir of virulence genes located on MGEs, and our clinical and experimental data indicated that ST22-MRSA is hypervirulent and has a strong ability to cause life-threatening diseases. Thus, the transmission of ST22-MRSA should be of concern. Furthermore, our findings are of great importance for elucidating the role of *agr* expression in CA-MRSA evolution. A major limitation of this study is that, due to a lack of available clinical records, we only identified 12 ST22 MRSA isolates as CA-MRSA in accordance with the Centers for Disease Control and Prevention definition among 749 MRSA strains from China between 2014 and 2019. This means that such a small sample size of the ST22-MRSA clone may result in the difficulty of evaluating the transmission tendency and the potential threat of this clone in China, both outside and within health care settings. Therefore, the findings about the ST22 clone from China should be interpreted with caution. Further studies will be required to clarify how the ST22 MSSA isolates from China evolved to obtain methicillin resistance and the virulence of ST22 MRSA isolates and ST22 MSSA isolates. Furthermore, we will also record as many clinical data as possible in the future data collection to distinguish community infection from hospital infection.

## MATERIALS AND METHODS

### Collection of bacterial isolates.

Thirty-three ST22 MRSA and 68 MSSA ST22 isolates were collected from patients with bloodstream infections in 18 provinces in China from 2014 to 2019. Among these ST22 S. aureus isolates, 21 were classified as CA according to the Centers for Disease Control and Prevention definition (12 CA-MRSA and 9 CA-MSSA). The antimicrobial susceptibilities of 21 ST22 isolates to 16 antimicrobial agents were determined using the agar dilution method in accordance with the Clinical and Laboratory Standards Institute protocols (25).

### Whole genome sequencing and genomic analysis of global ST22 clones.

Whole genome sequencing of the ST22 genomes was carried out on a HiSeq X 10-PE150 platform (Illumina, San Diego, CA, USA). SPAdes v3.14.1 (26) was used to assemble the processed Illumina data (after adaptor trimming and quality filtering). The genome annotation of ST22 isolates was performed with DFAST-core v1.2.11 (27). Genomes of additional 284 ST22 strains from other regions were downloaded from GenBank for comparison (Table S1).

### Phylogenetic analysis and Bayesian evolutionary analysis.

Snippy v4.6.0 (https://github.com/tseemann/snippy) was used to identify SNPs for the ST22 core genome; the S. aureus HO 5096 0412 genome (GenBank no. HE681097) served as a reference. Core single nucleotide polymorphisms (SNPs) from the recombination-free core-genome alignment with RAxML (28) (1,000 bootstrap replications) were used to construct the phylogenetic tree for ST22 isolates. BactDating v1.1 (29) estimated node dates of non-ST22-A isolates, using the output from Gubbins (30) and isolation dates of the ST22 strains as the input. The minimum spanning trees (MSTs) based on core SNP data were constructed using PHYLOViZ v2.0 (https://online.phyloviz.net/index).

### Comparison of antimicrobial resistance genes, virulence factors, pathogenicity islands, and prophages among ST22 strains.

Antimicrobial resistance genes and virulence factors were identified by ABRicate v1.0.0 (https://github.com/tseemann/abricate) based on CARD and VFDB databases with default settings. Pathogenicity islands and prophages were compared using BLAST, with sequences of those of S. aureus as queries.

### Virulence assays.

All animal experiments were approved by the Institutional Animal Care and Ethics Committee of the First Affiliated Hospital of Zhejiang University, School of Medicine. To evaluate the virulence potential of ST22-MRSA *in vivo*, mouse skin infection and the bloodstream infection models were established as previously described (31). Briefly, for the former, 6-week-old BALB/c-nude female mice were used for this study. Mice were inoculated with 100 μL sterile NaCl solution containing OD_600_ = 0.5 live bacterial cells by subcutaneous injection (each group injected 5 mice). Abscess length (L) and width (W) values were measured to calculate the size of skin abscesses, i.e., abscess size = L × W. We measured and recorded lesions on the skin of each mouse 2 days after injection using calipers, and the normal blank group of mice injected with NaCl solution alone was used as the negative control. For the latter, 6-week-old BALB/c female mice were used. Mice were inoculated with 100 μL sterile NaCl solution containing 2 × 10^8^ live S. aureus by caudal vein injection (each group injected 10 mice). The normal blank group of mice injected with NaCl solution alone was used as negative control. After inoculation, the physical condition of each mouse was monitored and recorded every day. When the first mouse infected with S. aureus strains died, we euthanized all remaining mice for each group. As hemolysis (lysis of erythrocytes) is a crucial determinant of S. aureus virulence, we accessed hemolytic capacity of S. aureus strains as previously described (31). ST22 bacterial cultures were cultivated in TSB for 9 h and centrifuged. The supernatant was incubated with rabbit red blood cells (RBCs) for 3 h at 37°C. Hemolytic capacity was determined by measuring the optical density at 540 nm using a Micro ELISA Autoreader. Purified water was used as positive control, and rabbit RBCs resuspended in NaCl solution were used as negative control.

### Quantitative reverse-transcription (qRT)-PCR.

The expression of *hla* and *agrC* in ST22 isolates was evaluated by qRT-PCR, with *gyrB* as an internal control. We chose S. aureus USA300_FPR3757 (ATCC BAA-1556) as a reference strain due to its high expression of these genes (32).

### Statistical analysis.

All assays were performed in triplicate independently. The difference in skin lesions between mice was examined using *q* test. Kaplan–Meier analysis was performed to evaluate differences in the survival rates of mice infected with S. aureus. A chi-squared test was used to analyze the results of the hemolytic test. Unpaired *t* test was used to analyze the qRT-PCR data. All data were analyzed using GraphPad Prism 9.0 software (GraphPad, San Diego, CA, USA). *P < *0.05 was considered as statistically significant.

### Data availability.

The whole-genome sequences of 21 ST22 S. aureus isolates from this study have been deposited in the GenBank database under BioProject accession no. PRJNA769995.
