# Supplementary material for: Comparison of Genetic Features and Evolution of Global and Chinese Strains of Community-Associated Methicillin-Resistant Staphylococcus aureus ST22
Source: Microbiol Spectr. 2022 Feb 9;10(1):e02037-21. doi: 10.1128/spectrum.02037-21 (PMC8881084; doi:10.1128/spectrum.02037-21)
Supplement: SUPPLEMENTAL FILE 4 — Supplemental material. Download SPECTRUM02037-21_Supp_4_seq14.pdf, PDF file, 2.5 MB [file spectrum02037-21_supp_4_seq14.pdf]

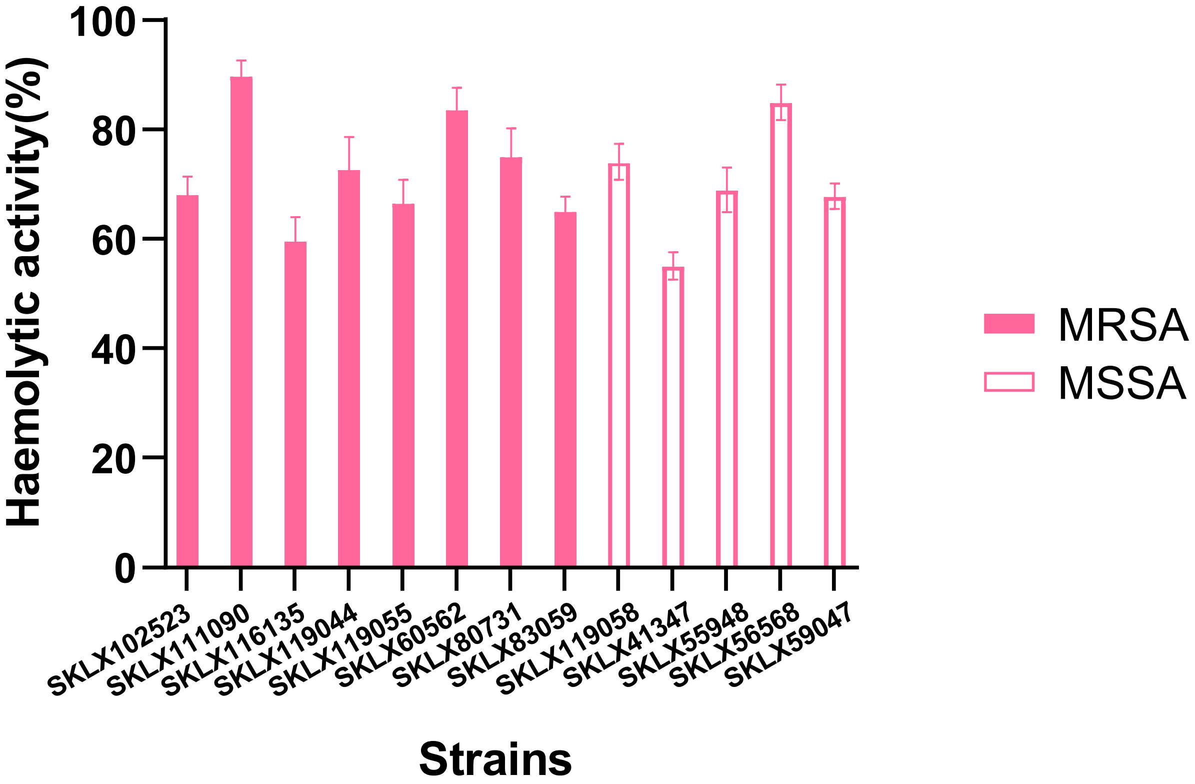

Figure S1. Haemolytic capacity estimates of selected isolates.

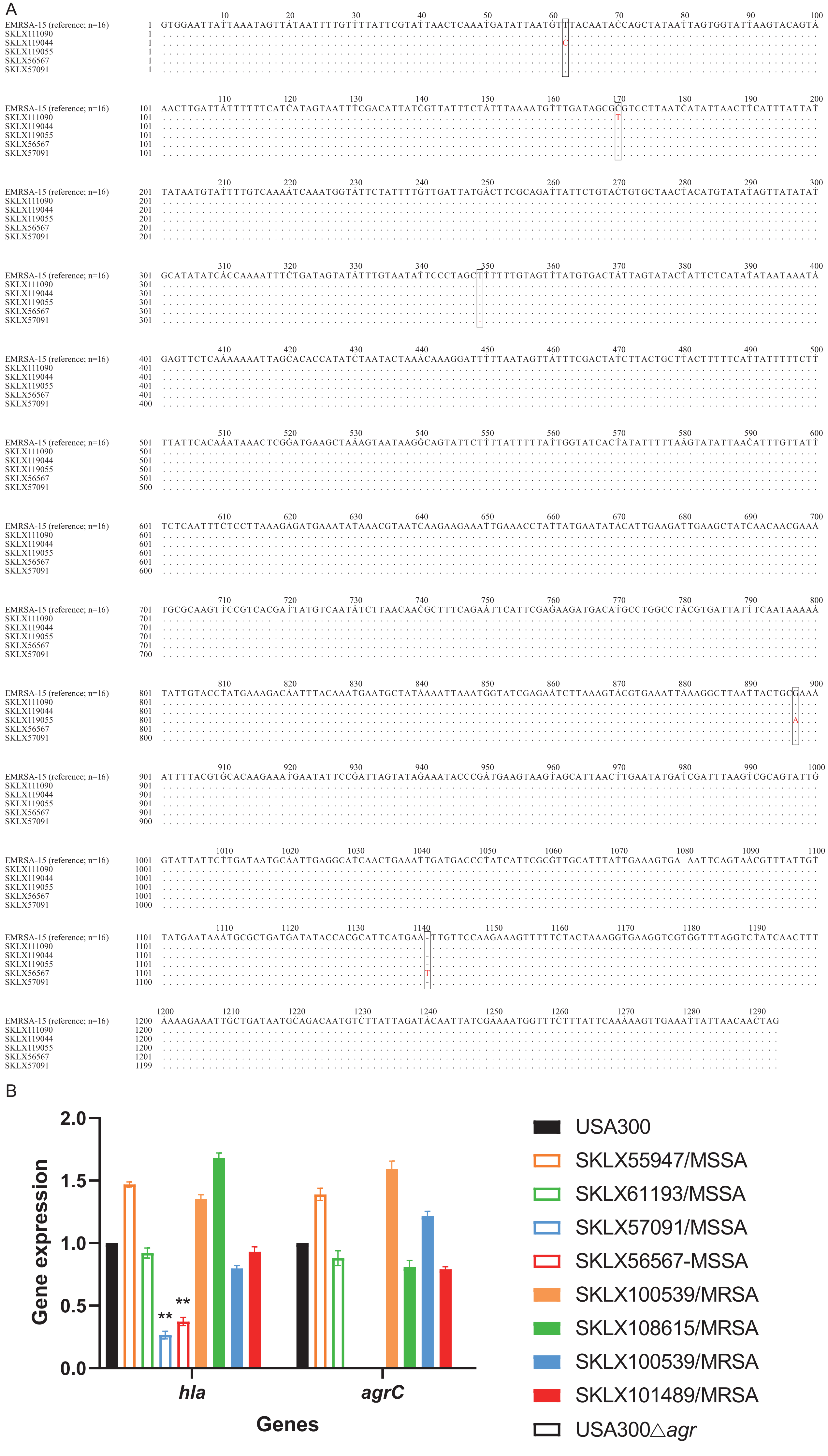

Figure S2. Alignment of the nucleotide sequence of *agrC* and expression of virulence toxins in ST22 isolates from China. (A) Sequence of the *agrC* gene in ST22 isolates from China compared to the reference sequence (*agrC* in EMRSA-15 strain HO 5096 0412). The dots indicate nucleotide residues identical to those of the reference sequence. Variable nucleotide residues were bounded by black boxes and highlighted by red. (B) Expression of *hla* and *agrC* in ST22 isolates compared to USA300. **P<0.01.
